# Supplementary material for: Prognostic Value and Clinical Significance of FGFR Genomic Alterations (GAs) in Metastatic Urothelial Cancer Patients
Source: J Clin Med. 2022 Aug 1;11(15):4483. doi: 10.3390/jcm11154483 (PMC9369263; doi:10.3390/jcm11154483)
Supplement: Supplementary file 1 [file jcm-11-04483-s001.zip › jcm-1808168-supplementary.pdf]

Table S1. Clinical and anatomopathological characteristics of 98 UC patients sequenced for FGFR alterations, including 77 cases with advanced disease (study population).

Variable	Modality	n = 98
**Age**	Mean ± sd	67 ± 10 [35 - 92]
**Sex**	Male	71 (72.4%)
	Female	27 (27.6%)
**ECOG PS**	0	37 (37.7%)
	1	31 (31.6%)
	2	2 (2.1%)
	Not available	28 (28.6%)
**Smoking**	Non smoker	19 (19.4%)
	Current smoker	15 (15.3%)
	Former smoker	46 (46.9%)
	Not available	18 (18.4%)
**FGFR**	Mutation/Fusion	31 (31.6%)
	WT	67 (68.4%)
**Tumor location**	Bladder	78 (79.6%)
	Non bladder	20 (20.4%)
**Surgery**	No	5 (5.1%)
	Yes	93 (94.9%)
**Days to Surgery**	Median (IQR)	24.00 (0.00 - 96.75)
**Surgery extension**	Cystectomy (RC)	52 (56%)
	Nephroureterectomy (NU) /Nephrectomy	15 (16.1%)
	NU + RC	5 (5.4%)
	Ureterectomy	1 (1%)
	TURBT	20 (21.5%)
**Lymphadenectomy**	No	41 (41.8%)
	Yes	53 (54.1%)
	Not available	4 (4.1%)
**Bladder Preservation**	Radiotherapy	4 (4.1%)
	Chemo-Radiotherapy	7 (7.1%)
	No	87 (88.8%)
**pT**	1	9 (9.2%)
	2	38 (38.8%)
	3	37 (37.8%)
	4	13 (13.2%)
	Not available	1 (1.0%)
**pN**	0	24 (45.3%)
	1	15 (28.3%)
	2	13 (24.5%)
	3	1 (1.9%)
**Number positive lymph nodes**	Median (IQR)	1.00 (0.00 -1.50)
	Not available	6 (11.3%)
**Number lymph nodes resected**	Median (IQR)	8.0 (5.0 - 11.5)
	Not available	10 (18.9%)
**Grade**	2	3 (3.1%)
	3	93 (94.9%)
	Not available	2 (2%)
**Histology**	Transitional cells	87 (88.8%)
	Squamous	7 (7.1%)
	Anaplastic	1 (1.0%)
	Neuroendocrine	1 (1.0%)

	Sarcomatoid	2 (2.1%)
**Perioperative Chemotherapy**	No	58 (59.2%)
	Neoadjuvant	17 (17.3%)
	Adjuvant	23 (23.5%)
**Chemotherapy schedule**	Cisplatin-based	32 (80%)
	Carboplatin-Gemcitabine	7 (17.5%)
	Durvalumab-Olaparib (Clinical Trial)	1 (2.5%)
**Days to Chemotherapy**	Median (IQR)	60 (33 - 114)
	Not available	58 (59.2%)
**Number of cycles administered**	Median (IQR)	3.5 (2.8 - 4.0)

Table S2. ORR according to the type of FGFR genomic alteration (mutation, fusion, amplification).

						**p value**
			**First line: Cisplatin (n = 13)**			
		**CR**	**PR**	**SD**	**PD**	**0.64**
**FGFR**	***Mutation***	1 (14.3%)	4 (57.1%)	2 (28.6%)	0 (0.0%)	
	Fusion	0 (0.0%)	3 (75.0%)	0 (0.0%)	1 (25.0%)	
	Amplification	0 (0.0%)	1 (50.0%)	1 (50.0%)	0 (0.0%)	
			**First line: Immunotherapy (n = 6)**			**p value**
		**CR**	**PR**	**SD**	**PD**	**1**
**FGFR**	***Mutation***	1 (33.3%)	1 (33.3%)	0 (0.0%)	1 (33.3%)	
	Fusion	0 (0.0%)	1 (50.0%)	0 (0.0%)	1 (50.0%)	
	Amplification	0 (0.0%)	0 (0.0%)	0 (0.0%)	1 (100.0%)	
			**First line: FGFR inhibitors (n = 5)**			**p value**
		**CR**	**PR**	**SD**	**PD**	**1**
**FGFR**	***Mutation***	0 (0.0%)	1 (25.0%)	0 (0.0%)	3 (75.0%)	
	Fusion	0 (0.0%)	0 (0.0%)	0 (0.0%)	1 (100.0%)	
	Amplification	0 (0.0%)	0 (0.0%)	0 (0.0%)	0 (0.0%)	
			**Second line: Immunotherapy (n = 8)**			**p value**
		**CR**	**PR**	**SD**	**PD**	**0.57**
**FGFR**	***Mutation***	0 (0.0%)	0 (0.0%)	1 (33.3%)	2 (66.7%)	
	Fusion	0 (0.0%)	1 (33.3%)	0 (0.0%)	2 (66.7%)	
	Amplification	0 (0.0%)	1 (50.0%)	1 (50.0%)	0 (0.0%)	
			**Second line: FGFR inhibitors (n = 11)**			**p value**
		**CR**	**PR**	**SD**	**PD**	**0.79**
**FGFR**	***Mutation***	1 (12.5%)	5 (62.5%)	1 (12.5%)	1 (12.5%)	
	Fusion	0 (0.0%)	1 (100.0%)	0 (0.0%)	0 (0.0%)	
	Amplification	0 (0.0%)	1 (50.0%)	0 (0.0%)	1 (50.0%)	
			**Third line: Immunotherapy (n = 8)**			**p value**
		**CR**	**PR**	**SD**	**PD**	**1**
**FGFR**	***Mutation***	1 (16.7%)	5 (83.3%)	0 (0.0%)	0 (0.0%)	
	Fusion	0 (0.0%)	1 (100.0%)	0 (0.0%)	0 (0.0%)	
	Amplification	0 (0.0%)	1 (100.0%)	0 (0.0%)	0 (0.0%)	

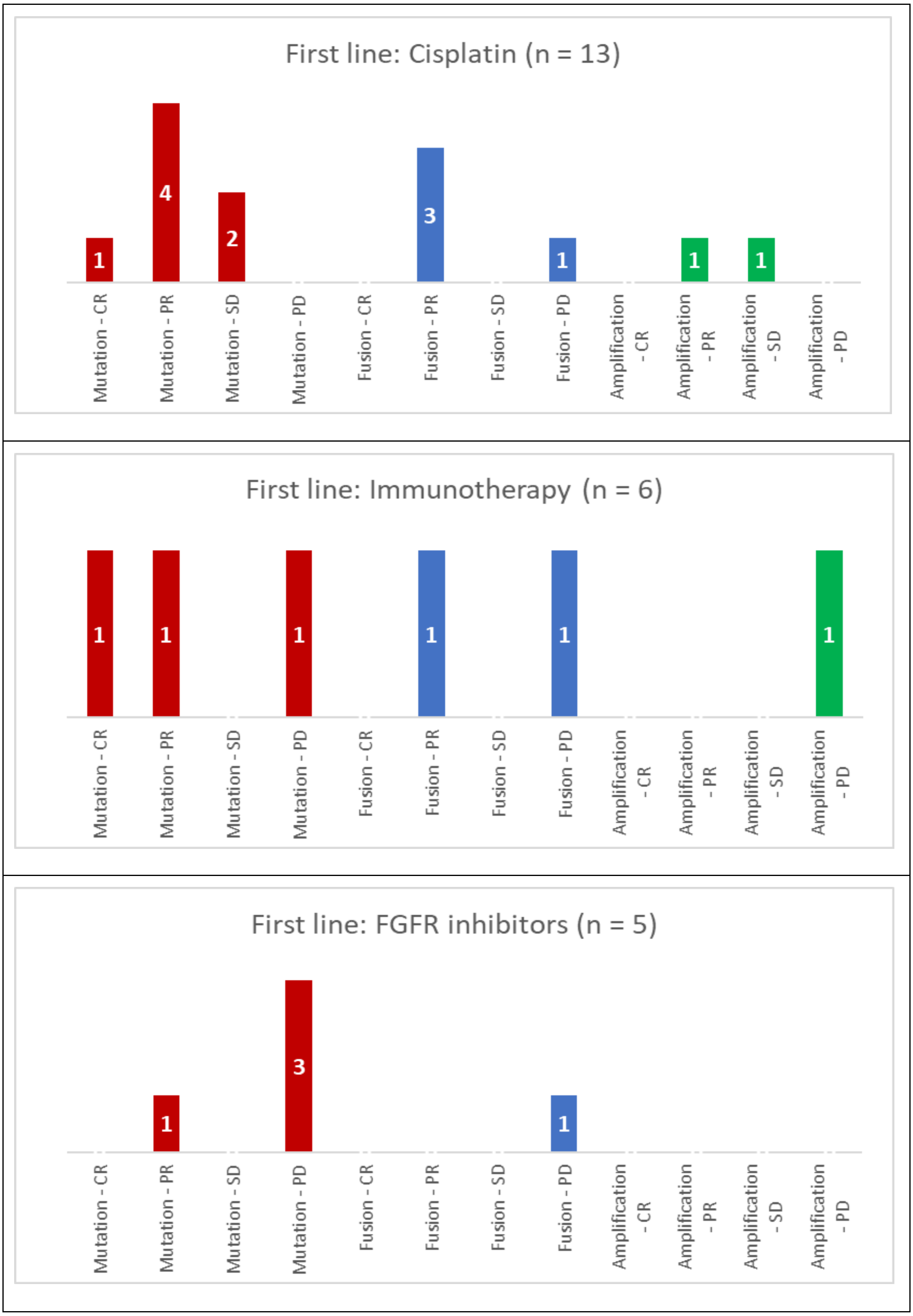
First line: Cisplatin (n = 13)
1
4
2
3
1
1
1
Mutation - CR
Mutation - PR
Mutation - SD
Mutation - PD
Fusion - CR
Fusion - PR
Fusion - SD
Fusion - PD
Amplification - CR
Amplification - PR
Amplification - SD
Amplification - PD
First line: Immunotherapy (n = 6)
1
1
1
1
1
1
Mutation - CR
Mutation - PR
Mutation - SD
Mutation - PD
Fusion - CR
Fusion - PR
Fusion - SD
Fusion - PD
Amplification - CR
Amplification - PR
Amplification - SD
Amplification - PD
First line: FGFR inhibitors (n = 5)
1
3
1
Mutation - CR
Mutation - PR
Mutation - SD
Mutation - PD
Fusion - CR
Fusion - PR
Fusion - SD
Fusion - PD
Amplification - CR
Amplification - PR
Amplification - SD
Amplification - PD

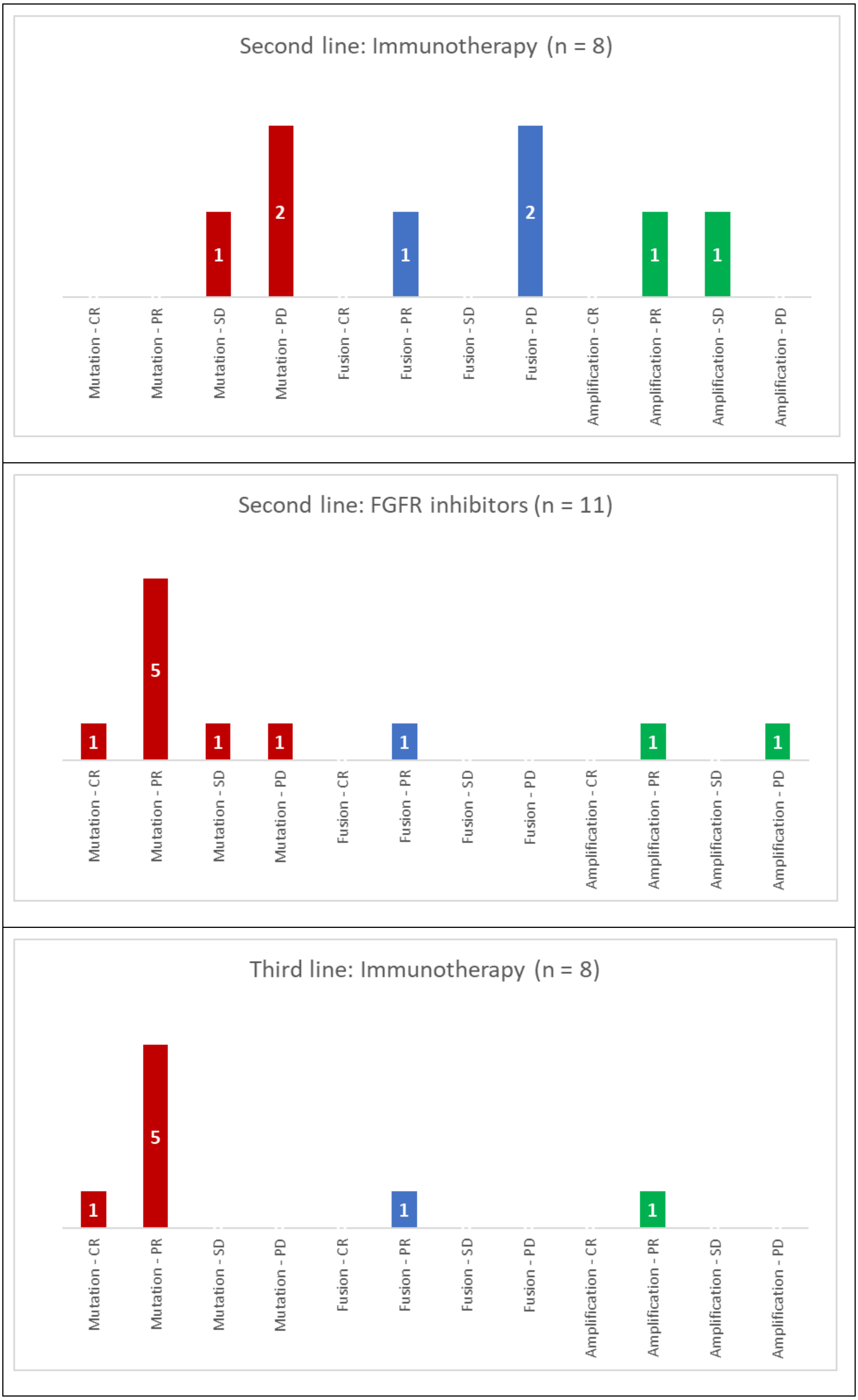

Figure S1. ORR according to the type of FGFR genomic alteration (mutation, fusion, amplification).

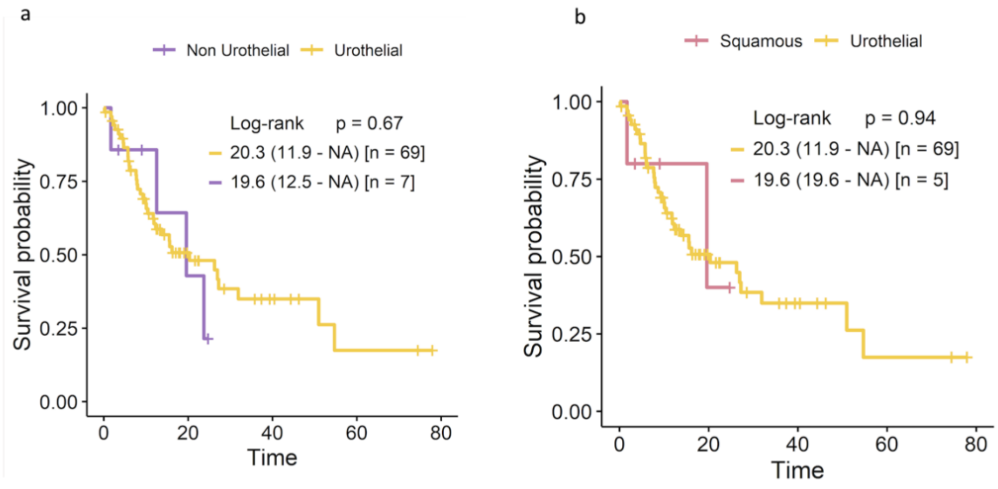

Figure S2:_OS according to the histology type: (a) urothelial vs non urothelial (b) squamous vs urothelial

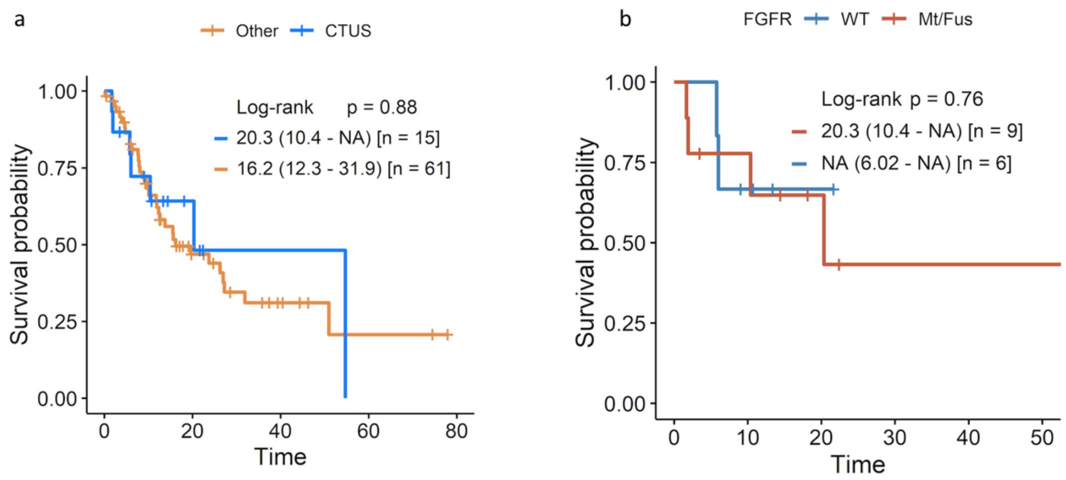

Figure S3: OS in UTUC in general population and stratified by FGFR status: (a) UTUC vs bladder (b) UTUC by FGFR status.

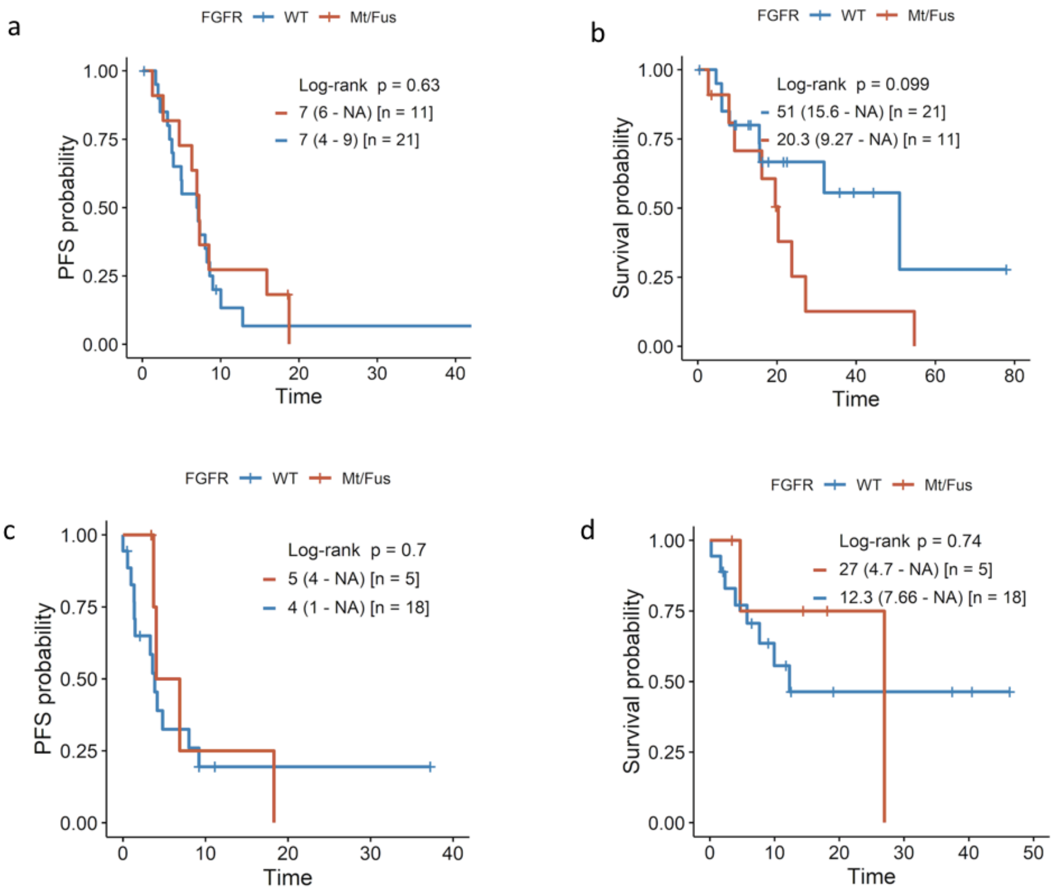

Figure S4. (**a**) PFS for tumors with FGFR GAs assessed from the exposition to platinum-based chemotherapy; (**b**) OS for tumors with FGFR GAs assessed from the exposition to platinum-based chemotherapy; (**c**) PFS for tumors with FGFR GAs assessed from the exposition to checkpoint inhibitors; (**d**) OS for tumors with FGFR GAs assessed from the exposition to checkpoint inhibitors.
